# Dissociation of mobile-bearing patellar component in low contact stress patellofemoral arthroplasty, its mechanism and management: two case reports

**DOI:** 10.1186/1757-1626-2-7502

**Published:** 2009-05-26

**Authors:** Hans-Peter W van Jonbergen, Dirk M Werkman, Alexander FW Barnaart

**Affiliations:** Department of Orthopaedic Surgery, Deventer HospitalNico Bolkesteinlaan 75, 7416 SE DeventerThe Netherlands

## Abstract

**Introduction:**

Isolated patellofemoral osteoarthritis can be treated with patellofemoral arthroplasty.

**Case presentation:**

We present two cases of isolated patellofemoral osteoarthritis treated with Low Contact Stress patellofemoral arthroplasty; in both cases the polyethylene mobile-bearing of the patellar component dissociated from the metal backing. One patient had a revision to a Richards patellofemoral prosthesis, and in the second patient the arthroplasty was converted to a total knee prosthesis because of clinically important femorotibial osteoarthritis.

**Conclusion:**

The possible failure mechanisms are described. We suggest avoiding the use of a metal-backed mobile-bearing patellar component due to the risk of dissociation.

## Introduction

In patients older than 55 years, isolated patellofemoral osteoarthritis occurs in 24% of women and in 11% of men radiographically [1]. Surgical treatment should be reserved for the minority of patients with incapacitating pain and functional limitations, and for whom non-operative modalities, such as weight reduction and physical therapy, have failed. Total knee replacement results in predictable and durable positive results. However, for a degenerative disease involving only one compartment, total knee replacement is probably an overly aggressive intervention. Patellofemoral arthroplasty is a successful treatment alternative for isolated patellofemoral osteoarthritis. Good results may be expected with appropriate use and consideration of reported indications and contraindications [2]. Long-term outcome is related to the progression or development of femorotibial osteoarthritis, malposition of the prosthesis, and, to a lesser extent, wear and/or loosening of the patellar component.

We report two cases of patients who experienced a dissociation of the mobile-bearing polyethylene patellar component following patellofemoral arthroplasty using the Low Contact Stress Patellofemoral Joint Replacement prosthesis.

## Case presentations

### Case report 1

**Figure 1.**
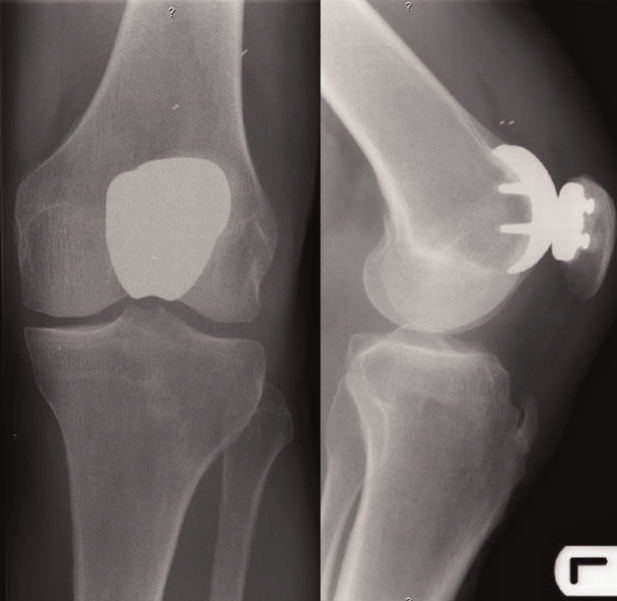
Radiograph of left knee showing dissociation of the polyethylene patellar component. The suprapatellar position of the polyethylene is demonstrated by the two metal markers visible just proximal to the trochlear component.

**Figure 2.**
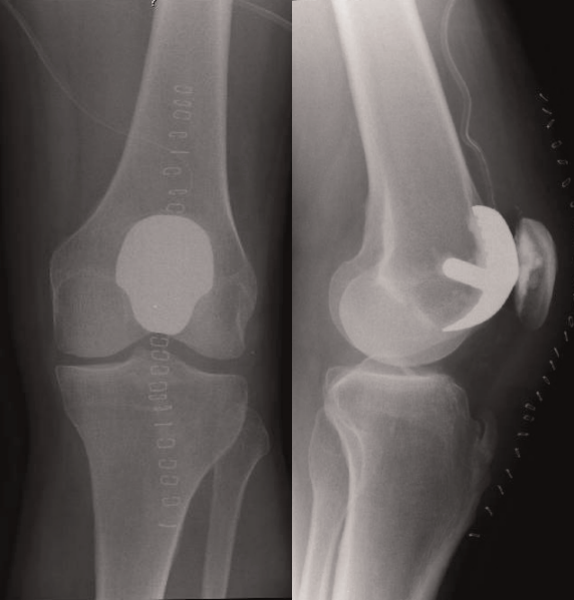
Radiograph of left knee after conversion to the Richards type II patellofemoral arthroplasty.

A 61-year-old Dutch Caucasian male patient presented with a 5-year history of left sided anterior knee pain with an increase in symptoms over the previous two years. On physical examination, there was full range of motion with no signs of patellofemoral instability, and radiographic studies demonstrated isolated degenerative changes of the patellofemoral compartment without signs of associated patellofemoral dysplasia. There was no history of knee surgery or trauma. Previous treatment with physical therapy had failed to alleviate symptoms. The patient underwent patellofemoral arthroplasty with the Low Contact Stress (LCS) Patellofemoral Joint Replacement prosthesis (DePuy Orthopaedics, Warsaw, Indiana) through a medial parapatellar incision. Stability (patellar tilt, subluxation) and impingement (catching) was tested over a full range of motion using trial implants before the definitive components were cemented in place. Immediate postoperative radiographs (anteroposterior and lateral non-weight bearing) confirmed adequate position of the prosthesis. Direct postoperative protected weight bearing with crutches was allowed, and he received antithrombotic prophylaxis with warfarin for eight weeks. During follow-up examinations at 2 weeks and 2 months postoperatively, normal healing was observed with 120 degrees of flexion. At three months postoperatively, the patient presented with subjective instability and audible crepitations and clicking. There was no history of recent injury or trauma. At 7 months postoperatively, he again presented with increasing pain and a mobile swelling proximal to the patella. Radiographs showed a displaced polyethylene liner of the patella (Figure 1). Revision of the patellofemoral arthroplasty was advised. During surgery the detached polyethylene liner was removed. The femoral component showed abrasive wear in the form of streaking due to articulation of the metal backing of the patellar component with the trochlear component. No signs of infection were identified. Both components were revised to a Richards type II patellofemoral prosthesis (Smith & Nephew, Memphis, Tennessee) (Figure 2). Postoperative rehabilitation was uneventful, and at last follow-up the patient demonstrated good function of the left knee with 120 degrees of flexion.

### Case report 2

**Figure 3.**
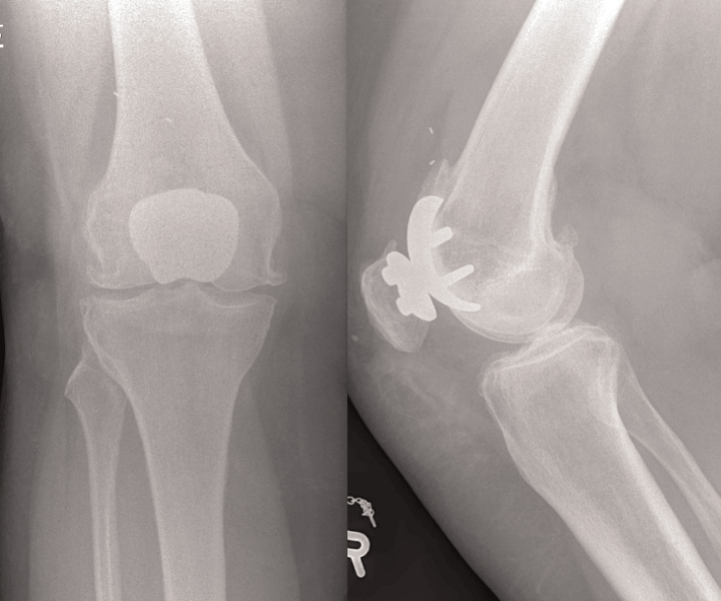
Radiograph showing dissociation of polyethylene patellar bearing. The femoral component of the LCS patellofemoral prosthesis is positioned in moderate flexion with increased thickness of the prosthesis-patella construct. The femorotibial compartment demonstrates osteophytes and narrowing of joint space.

A 55-year-old Dutch Caucasian woman was referred to our clinic with signs of dissociation of the polyethylene liner of a metal-backed patellar component in the right knee. Six years previously, she underwent an uneventful primary LCS patellofemoral arthroplasty with an uncemented patellar component. The patient reported a recent minor fall with direct injury to the anterior side of the knee, and later complained of persistent pain and noise arising from the knee while rising from a chair and climbing stairs. Walking on a level surface was uncomfortable with pain on both medial and lateral sides of the knee. Clinical examination showed no evident wasting of the quadriceps muscle, likely due to considerable obesity. Physical examination showed moderate valgus malalignment of both knees. No restriction of movement was observed with 0 to 130 degrees of flexion; however, a typical metal-on-metal grinding sound was heard from the anterior knee compartment during flexion and extension of the knee. There was significant tenderness on both medial and lateral femorotibial joint lines. Radiographs showed a displaced polyethylene liner of the patella with moderate femorotibial osteoarthritis (Figure 3). We advised a conversion to a total knee arthroplasty because of symptomatic femorotibial osteoarthritis. During surgery, the patellar button was found to be resting in synovial tissue in the suprapatellar pouch. No signs of infection were found, and no significant metallic synovitis was noted. Removal of the uncemented metal backing patellar component proved difficult with substantial loss of bone. After removal of the trochlear component, a NexGen (Zimmer, Warsaw, Indiana) total knee prosthesis was inserted with use of a cemented polyethylene patellar button. Postoperative rehabilitation is currently in progress. Cultures of synovial fluid were sterile.

**Figure 4.**
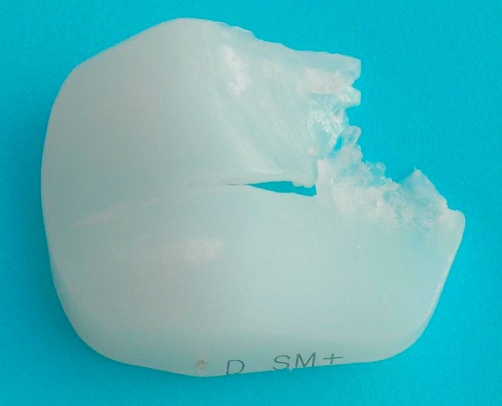
Photograph of the retrieved polyethylene patellar component. The fracture extends radially from the center of the specimen to the markedly thinned superolateral periphery.

The retrieved polyethylene patellar component showed a fracture radially extending from the center of the specimen to the markedly thinned superolateral periphery (Figure 4). This resulted in widening of the opening by which the polyethylene had been secured to the metal backing.

## Conclusion

Since the introduction of patellofemoral hemiarthroplasty by McKeever in 1949 [3], multiple designs of patellofemoral prosthesis have been introduced. Both the Lubinus and Richards patellofemoral resurfacing prostheses were introduced in the 1970s and made use of cemented trochlear and patellar components. In 1979 Blazina et al. noted concerning issues when using the Richards prosthesis, such as tracking of the patellar prosthesis when coming in and out of the trochlear groove proximally and distally [4]. The non-anatomic trochlear component is highly constrained with a deep central groove, and the polyethylene patellar component has a longitudinal ridge. Suggestions were made to lessen the deep femoral groove and adapt the shape of the patellar component [4]. Despite these concerns, the prosthesis has since been widely used although the number of reoperations for patellar maltracking in reported series is high [5,6].

To improve on the results of the Richards prosthesis and to avoid the high rate of patellar revisions, Merchant designed a modular patellofemoral prosthesis [7]. The patellar component was based on the successful Low Contact Stress Total Knee System, and the new trochlea incorporated some design features of the Richards prosthesis, such as a comparable sulcus angle. The patella could be used interchangeably with a femoral component of the LCS total knee prosthesis, obviating the need for revision of the patella. The metal-backed, mobile-bearing, self-aligning patellar component uses broad congruent articular surfaces for area contact loading. This is different from the more common point or line loading as seen with dome-shaped patellar components. In 2004 Merchant reported the early clinical results of the Low Contact Stress Patellofemoral Joint and reported good clinical outcome in 14 of 15 patients using the ADL scale [8]. However, the need for a mobile-bearing patellar component is debatable, as loosening and wear are not major reasons for revision of a patellofemoral arthroplasty [2,6]. Wear of the patellar polyethylene is although frequently seen in cases of revision for other reasons.

The reason for dissociation of the polyethylene component in the cases reported here remains uncertain. Failure of the metal-backed polyethylene patellar components in total knee arthroplasty has been well documented in the literature [9,10]. Several factors contribute to this type of failure. Specific implant related factors include the thickness of the polyethylene, the method that is used to attach the polyethylene to the metal, and the proportion of polyethylene that is backed by metal. Technique related factors, such as faulty patellar tracking and excessive thickness of the prosthesis-patella construct, might lead to increased stress on the polyethylene with resulting fatigue failure. Finally, patient related risk factors include obesity, good results in terms of high flexion, and relatively young age [9,10].

In our first patient, dissociation probably occurred at three months postoperatively. Also, the retrieved specimen was not fractured and showed no signs of wear. Given the fact that malposition with catching and locking is one of the main modes of failure in patellofemoral arthroplasty, it is most likely that the polyethylene liner was hooked and dislodged. Because neither of our patients had a history of patellofemoral instability prior to arthroplasty, unrecognized surgical prosthetic malalignment, especially rotation of the femoral component, could have led to patellar subluxation and catching. The operative records do not describe instability before cementing the definitive components in place, and the postoperative radiographs show adequate position of the prosthesis. In the second patient, the wear and subsequent fracturing and dissociation at 6 years postoperatively may be related to a combination of factors, including obesity, faulty patellar tracking, mild flexion of the femoral component, and overstuffing of the anterior compartment (Figure 3). The location of the marked thinning of the polyethylene in the superolateral quadrant further suggests that high shear stress occurs when the patella rides out of the intercondylar notch up into the trochlear groove of the femoral prosthesis during extension.

In the first patient we revised the patellofemoral prosthesis to the Richards type II prosthesis, which we have used in more than 200 patients since 1976. No technical difficulties were encountered during revision. In the second patient revision of the uncemented patellar component resulted in a substantial loss of bone, thereby making the revision difficult. Because of clinically important degenerative changes in the femorotibial compartments, a total knee prosthesis was inserted. The results of conversion to a total knee arthroplasty are comparable to the results of primary total knee arthroplasty [11,12].

We believe that the patellar component in patellofemoral arthroplasty should be compatible with current total knee designs. However, based on our experiences with the LCS Patellofemoral Joint Replacement, we suggest avoiding the use of a metal-backed, mobile-bearing patellar component. In addition to the risk of dissociation, revision of the non-cemented component may result in substantial loss of bone. Furthermore, the LCS Patellofemoral Joint Replacement patellar component is only compatible with the LCS total knee prosthesis. We consider a symmetrical dome-shaped cemented polyethylene component the best choice.

## List of abbreviations

ADL: Activity of Daily living
LCS: Low Contact Stress
